# A photodynamic pathway to apoptosis and necrosis induced by dimethyl tetrahydroxyhelianthrone and hypericin in leukaemic cells: possible relevance to photodynamic therapy

**DOI:** 10.1038/sj.bjc.6690066

**Published:** 1999-02

**Authors:** G Lavie, C Kaplinsky, A Toren, I Aizman, D Meruelo, Y Mazur, M Mandel

**Affiliations:** Institute of Hematology, Blood Transfusion Center, Tel-Hashomer, 52621, Israel; Department of Pathology, NYU Medical Center, New York, NY, 10016, USA; Department of Pediatric Hemato-oncology, Sheba Medical Center, Tel-Hashomer, 52621, Israel; Department of Hematology, Sheba Medical Center, Tel-Hashomer, 52621, Israel; Department of Organic Chemistry, The Weizmann Institute of Science, Rehovot, 76100, Israel

**Keywords:** photodynamic therapy, hypericin, dimethyl tetrahydroxyhelianthrone, Bcl-X, Bax, lamin, apoptosis

## Abstract

The mechanism of cell death induction by dimethyl tetrahydroxyhelianthrone (DTHe), a new second-generation photodynamic sensitizer, is analysed in human leukaemic cell lines in comparison with the structurally related hypericin. DTHe has a broad range of light spectrum absorption that enables effective utilization of polychromatic light. Photosensitization of HL-60 cells with low doses of DTHe (0.65 μM DTHe and 7.2 J cm^−2^ light energy) induced rapid apoptosis of ≥90% of the cells. At doses ≥2 μM, dying cells assumed morphological necrosis with perinucleolar condensation of chromatin in HL-60 and K-562 cell lines. Although nuclear fragmentation that is characteristic to apoptosis was prevented, DNA digestion to oligonucleosomes proceeded unhindered. Such incomplete apoptosis was more prevalent with the related analogue hypericin throughout most doses of photosensitization. Despite hypericin being a stronger photosensitizer, DTHe exhibited advantageous phototoxic properties to tumour cells, initiating apoptosis at concentrations about threefold lower than hypericin. Photosensitization of the cells induced dissociation of the nuclear envelope, releasing lamins into the cytosol. DTHe also differed from hypericin in effects exerted on the nuclear lamina, causing release of an 86-kDa lamin protein into the cytosol that was unique to DTHe. Within the nucleus, nuclear envelope lamin B underwent covalent polymerization, which did not affect apoptotic nuclear fragmentation at low doses of DTHe. At higher doses, polymerization may have been extensive enough to prevent nuclear collapse. Hut-78, CD4^+^ cells were resistant to the photodynamically activated apoptotic pathway. Beyond the tolerated levels of photodynamic damage, these cells died exclusively via necrosis. Hut-78 cells overexpress Bcl-X_L_ as well as a truncated Bcl-X_L_^tr^ isoform that could contribute to the observed resistance to apoptosis. © Cancer Research Campaign


British Journal of Cancer (1999) **79**(3/4), 423–432
© 1999 Cancer Research Campaign
Article no. bjoc.1998.0066

### A photodynamic pathway to apoptosis and necrosis induced by dimethyl tetrahydroxyhelianthrone and hypericin in leukaemic cells: possible relevance to photodynamic therapy

**G Lavie[1,2], C Kaplinsky[3], A Toren[3], I Aizman[4], D Meruelo[2], Y Mazur[5] and M Mandel[1,3]**

[1]Institute of Hematology, Blood Transfusion Center, Sheba Medical Center, Tel-Hashomer, 52621, Israel; [2]Department of Pathology, NYU Medical Center, New York, NY 10016, USA; [3]Department of Pediatric Hemato-oncology, and [4]Department of Hematology, Sheba Medical Center, Tel-Hashomer, 52621 Israel; [5]Department of Organic Chemistry, The Weizmann Institute of Science, Rehovot 76100, Israel

**Summary** The mechanism of cell death induction by dimethyl tetrahydroxyhelianthrone (DTHe), a new second-generation photodynamic sensitizer, is analysed in human leukaemic cell lines in comparison with the structurally related hypericin. DTHe has a broad range of light spectrum absorption that enables effective utilization of polychromatic light. Photosensitization of HL-60 cells with low doses of DTHe (0.65 $\mu$M DTHe and 7.2 J cm$^{-2}$ light energy) induced rapid apoptosis of ≥90% of the cells. At doses ≥2 $\mu$M, dying cells assumed morphological necrosis with perinucleolar condensation of chromatin in HL-60 and K-562 cell lines. Although nuclear fragmentation that is characteristic to apoptosis was prevented, DNA digestion to oligonucleosomes proceeded unhindered. Such incomplete apoptosis was more prevalent with the related analogue hypericin throughout most doses of photosensitization. Despite hypericin being a stronger photosensitizer, DTHe exhibited advantageous phototoxic properties to tumour cells, initiating apoptosis at concentrations about threefold lower than hypericin. Photosensitization of the cells induced dissociation of the nuclear envelope, releasing lamins into the cytosol. DTHe also differed from hypericin in effects exerted on the nuclear lamina, causing release of an 86-kDa lamin protein into the cytosol that was unique to DTHe. Within the nucleus, nuclear envelope lamin B underwent covalent polymerization, which did not affect apoptotic nuclear fragmentation at low doses of DTHe. At higher doses, polymerization may have been extensive enough to prevent nuclear collapse. Hut-78, CD4$^+$ cells were resistant to the photodynamically activated apoptotic pathway. Beyond the tolerated levels of photodynamic damage, these cells died exclusively via necrosis. Hut-78 cells overexpress Bcl-X$_L$ as well as a truncated Bcl-X$_L^{tr}$ isoform that could contribute to the observed resistance to apoptosis.

**Keywords:** photodynamic therapy; hypericin; dimethyl tetrahydroxyhelianthrone; Bcl-X; Bax; lamin; apoptosis

Photodynamic therapy (PDT), involving incorporation of photosensitizing molecules into malignant tumours and their destruction after excitation with light, offers an alternative treatment to conventional therapies, and is becoming increasingly accepted as a therapeutic modality in oncology (Dougherty, 1983; McCaughan, 1984). In addition to solid tumours, hairy-cell leukaemia and mycosis fungoides have also been treated with 8-methoxypsoralen and UVA (PUVA) (Honigsmann, 1987). The leading, most widely investigated, photosensitizers in PDT are the haematoporphyrins (HPD-Photofrin II) (Kessel, 1984), which were recently approved for clinical use. However, their use may be limited because of prolonged cutaneous phototoxicity, aggregation tendency, slow metabolism in vivo (Gomer, 1991; Grossweiner, 1994; Jones et al, 1996), as well as limited efficacies in affecting penetrating solid tumours (Orenstein et al, 1996). To overcome these limitations, a number of photosensitizing molecules are being investigated as potential agents for use in PDT. Such molecules are expected to exhibit intrinsic properties of absorption in the visible range of the spectrum, preferentially in the long range, to generate high singlet oxygen yield and to concentrate effectively within tumours. Polycyclic quinones (PQ) appear to possess some of these properties and we compare the activities of two highly phototoxic PQ in vitro.

*Received 11 August 1997*
*Revised 14 May 1998*
*Accepted 21 May 1998*

*Correspondence to:* G Lavie, Blood Transfusion Center, Sheba Medical Center, Tel-Hashomer 52621, Israel

Hypericin (HY), a polycyclic aromatic ketone with a meso-naphthodianthrone chromophore, is one example of a photosensitizer that can act by a variety of mechanisms. HY generates a high quantum yield of singlet oxygen (Thomas et al, 1992), superoxide anions (Hadjur et al, 1994) and semiquinone radicals (Weiner and Mazur, 1992; Diwu and Lown, 1993). It was shown to be cytotoxic to fibroblasts (Hadjur et al, 1995) and mammary carcinoma cells in vitro (Thomas and Pardini, 1992). HY binds well to tumour cells in vivo and is retained within tumours for longer periods than in normal tissues (Chung et al, 1994). The molecule also possesses inhibitory activities of cell proliferation, signal transduction pathways. HY was shown to inhibit protein kinase C (PKC), particularly when PKC translocates to the cell membrane after cell activation (Takahashi et al, 1989). HY is under evaluation as a tumoristatic agent in brain glioblastoma because of its PKC inhibitory activity. PKC appears to play an important role in signal transduction of glioblastoma cell proliferation (Anker et al, 1995). HY acts as inhibitor of mitogen-activated protein (MAP) kinase (Agostinis et al, 1995) and epidermal growth factor receptor tyrosine kinase (Agostinis et al, 1996). HY was found to be a virucidal agent (Meruelo et al, 1988; Lavie et al, 1989; Tang et al, 1990) because of its photodynamic properties (Carpenter and Kraus, 1991; Degar et al, 1993; Hudson et al, 1993).

Efforts to identify novel efficacious agents for photodynamic therapy led us to evaluate the photodynamically induced cytotoxicities of a newly designed photosensitizer 10,13-dimethyl 1,3,4,6-tetrahydroxyhelianthrone (DTHe) (Figure 1), in leukaemic cell lines. DTHe was chosen because of its dibenzperylenequinone chromophore (seven aromatic rings) that has absorption spectral properties virtually identical to hypocrellins, potent anti-tumoral photosensitizers isolated from the parasitic fungus *Hypocrella bambuase* which grows in China and Tibet (Diwu, 1990; Diwu, 1995; Miller, 1997). DTHe also shares structural similarities with HY and was anticipated to be a potent photosensitizer owing to its considerable light absorbance in the visible range of the spectrum. The phototoxicity profiles and mechanisms of cell death induction of DTHe were analysed in comparison with those of HY in HL-60, K-562 and Hut-78 leukaemic cell lines to evaluate their potential for further development for clinical utilization.

### MATERIALS AND METHODS

### Preparation of hypericin

Hypericin, (HY) 10,11-dimethyl-1,3,4,6,8,13-hexahydroxy-naphthodianthrone was synthesized by self condensation of emodin anthrone (Lavie et al, 1990). Emodin anthrone (Societa Inverni della Beffa, Milano, Italy) dissolved in pyridine solution was heated with piperidine, pyridine-*N*-oxide and catalytic amounts of ferrous sulphate (Aldrich Chemicals). The resulting protohypericin was irradiated with visible light to yield free HY. Crude HY obtained after concentration and trituration with aqueous hydrochloric acid was crystallized from pyridine, resulting in HY–pyridine complex, which was heated to 160°C for 2 h under high vacuum to form free HY. Free HY was dissolved in methanol and converted to its monosodium salt by adding aqueous sodium hydrogen carbonate followed by precipitation with hexane and crystallization from methanol. The compound was purified by chromatographies on silica gel (Merck 60, 70–230 µm mesh) using as a mobile phase methanol/ethanoic acid (2:1) and aqueous sodium dihydrogen phosphate (1%), to a degree of purity of 99.7%. This was determined by high-performance liquid chromatography (HPLC) with a diode array detector and a Merck Lichrosorb column RP 18.5 µm. HY was dissolved in 70% aqueous ethanol to a stock solution of 4 mM from which subsequent dilutions were made in the cell culture media to obtain a final ethanol concentration of 1%.

### Preparation of 10,13-dimethyl-1,3,4,6- tetrahydroxy-helianthrone (DTHe)

1,3-dihydroxy-7-methyl anthraquinone was converted to the respective anthrone by reflux in acetic acid containing hydrochloric acid and tin chloride. The crude anthrone was dissolved in ethanol and treated with a solution of ferric chloride in ethanol to yield a bianthrone, which was then dissolved in aqueous ammonia and heated to 100°C while a strong stream of air was passed through the solution. The product was isolated by acidification of the solution and extraction with ethanoic acid followed by silica gel chromatographies. DTHe was eluted with acetone/hexane (6:4) and crystallized from a mixture of the two solvents. A purity of 97.5% was established by HPLC as described above using as the mobile phase methanol:EtOAc (2:1) and aqueous sodium dihydrogen phosphate (1%). DTHe was dissolved in 70% aqueous ethanol to a stock solution of 4 mM and further diluted as above.

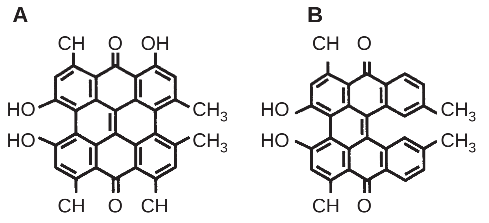

**Figure 1** The chemical structures of hypericin (**A**) and dimethyl tetrahydroxyhelianthrone (DTHe) (**B**)

### Singlet oxygen generation

Quantum yields of singlet oxygen were determined by a method based on calculation of the primary fractions of the sensitizers and singlet oxygen as a function of diphenyl isobenzofuran (DPBF) concentration, using pulse laser (Chattopadhyay, 1984). The method involves monitoring the negative change of optical density $-\Delta(OD)^{\lambda 1}$, due to total DPFB depletion, and the positive end of pulse change in optical density, due to benzophenone triplet formation in benzene, $\Delta(OD)^{\lambda 2}_{o}$ at their absorption maxima. HY and DTHe were dissolved in both ethanol and liposomes to $10^{-5}$ M. Laser flash photolysis was carried out using a 337.1-nm nitrogen AVCO Everett laser with a pulse duration of 10 ns and 0.5 nm half-bandwidth. A solution of benzophenone in benzene was used as actinometer in flash photolysis experiments having the same optical density at excitation wavelength; excitation coefficient of T–T absorption at 532 nm being $7.6 \times 10^3$ $M^{-1}$ $cm^{-1}$.

### Cell lines

Human HL-60 promyelocytic leukaemia cells, K-562 and Hut-78, $CD4^+$ T-cells were obtained from the American Tissue culture collection (ATCC). HL-60 and Hut-78 cells were grown in RPMI-1640 supplemented with 15% fetal calf serum, 100 mM glutamine and 100 U $ml^{-1}$ penicillin–streptomycin. K-562 human erythroleukaemia cells were grown in RPMI-1640 supplemented with 10% fetal calf serum. All cell lines were cultured in a humidified 5% carbon dioxide/95% air atmosphere at 37°C.

### Cell viability

Cell viability was monitored by the MTT assay which measures formation of formasan from 3-(4,5-dimethylthiazol-2-yl)-2,5-diphenyl tetrazolium bromide in mitochondria of viable cells as described previously by Mossman (1983). Cells were plated in flat-bottomed 96-well plates at $10^5$ cells per well in 200 µl of medium that contained the photodynamic reagents. MTT was added 16 h after photosensitization, incubated with the cells for 3 h and analysed in an enzyme-linked immunosorbent assay (ELISA) reader at 560 nm. Corrections were made for non-specific absorption of MTT in medium in the absence and presence of HY or DTHe at each dose level. Cell viability of ≥95%, by Trypan blue exclusion from 400 cells, was a prerequisite in each experiment.

### Photodynamic excitation

Light irradiation was performed from a source of two parallel 40-W fluorescent tubes placed at a fixed distance of 16 cm from the culture plates and measured to emit polychromatic white light at a

 © Cancer Research Campaign 1999

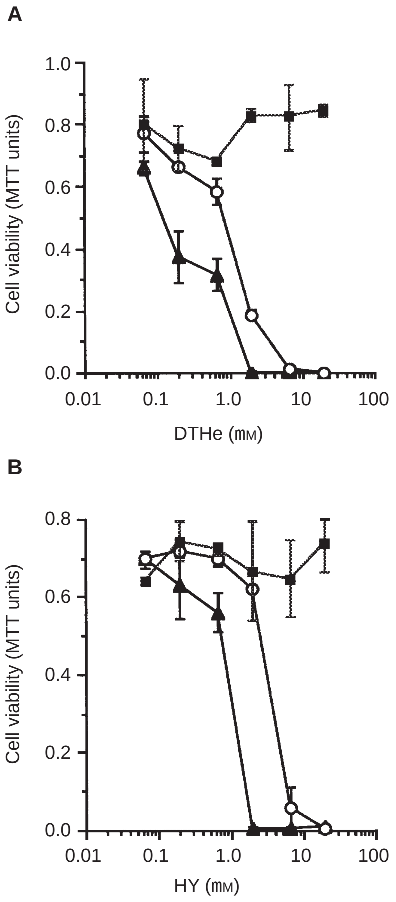

**Figure 2** The effects of reagent concentrations and light dose on HL-60 cell viability in an MTT assay. Cells were plated in triplicate, $10^5$ cells per well, in 100 µl of medium in 96-well microplates. HY and DTHe were added from 2 × concentrations in growth medium (prepared from concentrated stock solutions in 70% ethanol and yielding a final ethanol concentration of 1% in medium) resulting in 200 µl in final concentrations ranging from 0.65 to 20 µM with 0.5 $\log_{10}$ dose increments. Immediately thereafter, the cells were subjected to irradiation from a fluorescent source at a fluence rate of 4 mW $cm^{-2}$ for 0 (■), 20 (○) (4.8 J $cm^{-2}$) and 60 (▲) min (14.4 J $cm^{-2}$). The cells were then incubated for 16 h in a 37°C, 5% carbon dioxide incubator, the medium removed carefully (150 µl) and the MTT reagent added for 4 h. Three repetitions of the experiment were performed fluence rate of 4 mW $cm^{-2}$ in wavelengths that ranged between 380 and 700 nm. Light intensities were quantitated using the IL 1350 Radiometer/Photometer, from International Light.

### Determination of percentage of apoptotic cells

Percentage of apoptotic cells was determined by light microscopy on cytospin cell preparations stained with May–Grunwald–Giemsa according to the method described previously (Lotem et al, 1991, 1995). Apoptotic cells were recognized by their smaller size and nuclei fragmented into condensed chromatin bodies. The results were validated against a standard induction of apoptosis by heat treatment at 43°C for 40 min followed by incubation for 4 h at 37°C according to the method described by Li et al (1994). One hundred cells in four preparations (total of 400 cells) were counted by two individuals, independently. The data are given as the means and standard deviations calculated per four events. Morphological necrosis was recognized by loss of basophilic staining from the cytoplasm and nuclei, formation of pale chromatin and heavily stained perinucleolar rings of condensed chromatin.

### Flow cytometry analyses

Cells harvested 5 h after administration of photodynamic stress were rinsed with phosphate-buffered saline (PBS) and fixed with 70% aqueous ethanol. The cells were then resuspended in phosphate–citrate buffer (PC buffer) pH 7.8 (192 parts of 0.2 M disodium hydrogen phosphate and eight parts of 0.1 M citric acid) at room temperature for 30 min and stained with propidium iodide in PC buffer containing 10 µg $ml^{-1}$ RNAase A. The cells were then analysed in a Coulter EPICS XL-MCL flow cytometer with the entire field gated.

### DNA fragmentation assay

DNA degradation to oligonucleosomes, that is characteristic to cells undergoing apoptosis, was assayed as described previously (Lotem and Sachs, 1995). Cells ($2 \times 10^6$) pelleted in Eppendorf tubes were lysed in 0.5 ml lysis buffer containing 10 mM tris-HCl, pH 7.5, 0.6% sodium dodecyl sulphate (SDS), 10 mM EDTA and 15 µg $ml^{-1}$ RNAase mixture (Ambion, Austin, TX, USA). After incubation at 37°C for 20 min, sodium chloride was added to 1 M and the mixture was kept overnight at 4°C. The preparation was spun at 14 000 ***g*** for 30 min at 4°C, the supernatant collected, phenol extracted and DNA precipitated overnight at –20°C by adding 1 ml of ethanol. The DNA pellet was air-dried, dissolved in 20 µl TE buffer (10 mM Tris, 10 mM EDTA, pH 7.5) at 4°C for 24 h, electrophoresed for 4 h at 2 V $cm^{-1}$ in 1.2% agarose gel containing 0.5 µg $ml^{-1}$ ethidium bromide and photographed under UV light.

### Western blots

Cells were lysed in 50 mM Tris, 50 mM sodium chloride buffer, pH 8.0, that contained 1% Triton X-100 and a Complete Protease Inhibitor Cocktail, 40 µl $ml^{-1}$ (Boehringer Mannheim, Germany) for 15 min. The nuclei were pelleted at 2500 ***g*** for 10 min at 4°C. Samples that consisted of the cytosolic fraction were loaded onto 10–12.5% SDS-PAGE minigel, 20 µg protein per lane. The nuclear fractions, $10^6$ cells per lane, were lysed in Laemmli buffer, electrophoresed on gels and transblotted to nitrocellulose filters. Each filter was reacted with one of the following: anti-Bcl-2 mouse monoclonal antibody (clone no. 124, Dako, Glostrup, Denmark), anti-Bcl-x (rabbit polyclonal antibody S-18, Santa Cruz Biotechnology, CA, USA) raised against a synthetic peptide that corresponds to amino acids 2–19 of human bcl-x and anti-Bax (rabbit polyclonal antibody 20, Santa Cruz). Anti-human actin antibody (mouse monoclonal clone no. 4, Dako) was used as a reference to standardize cytosolic extracts. Specificity of interaction with Bcl-x was verified by competition with a synthetic peptide that corresponds to N-terminus amino acids 2–19 of human Bcl-x. Corresponding peroxidase-tagged second antibodies (goat anti-rabbit or anti-mouse) were used and the blots developed in the BM chemiluminescence kit from Boehringer Mannheim according to the manufacturer's instructions.

### RESULTS

### Photodynamic effects of HY and DTHe on HL-60 cell viability

The phototoxicities of DTHe and HY to HL-60 cells were compared after exposures to two doses of light irradiation: 4.8 or

© Cancer Research Campaign 1999

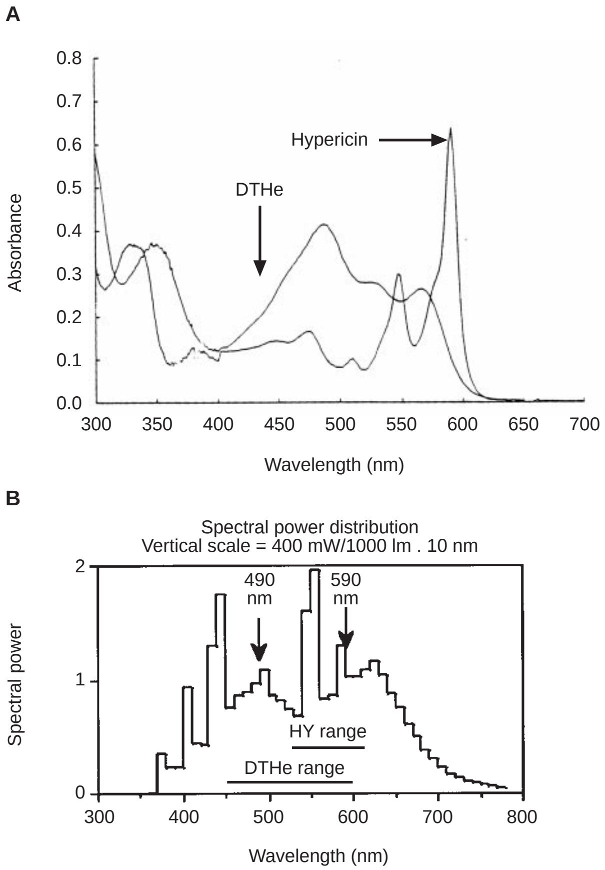

**Figure 3** (**A**) The absorption spectra of 0.01 mM DTHe and HY solubilized in ethanol. (**B**) The emission spectrum of the polychromatic fluorescent light source, 72 Biolux, used for photosensitization. Spectral power distribution data were obtained from the manufacturer's catalogue of OSRAM, Munich, Germany

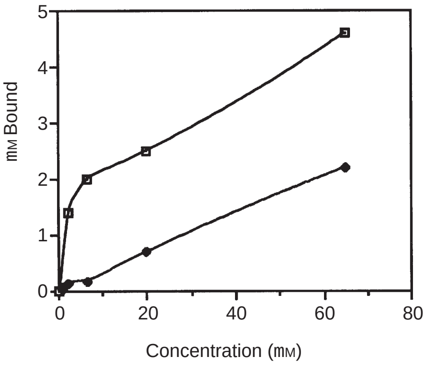

**Figure 4** Comparison of the cell binding properties of HY (⊡) and DTHe (●). HL-60 cells, 2×10$^6$ in 1 ml growth medium, were administered with 0, 0.65, 2, 6.5, 20 and 65 μM of either HY or DTHe in triplicates. The cultures were incubated at 37°C in a 5% carbon dioxide incubator for 1 h in the dark. The cells were washed 3× with PBS and lysed in distilled water containing 0.4% Triton X-100. The nuclei were spun down at 2000 ***g*** for 10 min. The optical densities of the supernatants were determined at 590 nm for HY and at 490 nm for DTHe and plotted against calibration curves of HY and DTHe established in distilled water containing 0.4% Triton X-100

14.4 J cm$^{-2}$, obtained by irradiation for 20 or 60 min respectively. Cell viability was monitored after 16 h by the MTT assay. The results suggest that DTHe (Figure 2A) exhibits a more potent phototoxic activity in comparison with HY (Figure 2B). Cell death with DTHe occurred with an $LD_{50}$ of 1 μM at 4.8 J cm$^{-2}$, which was repeatedly observed to be about threefold lower than that of HY (3 μM). A more potent phototoxic activity of DTHe was also seen at the higher light dose of 14.4 J cm$^{-2}$ ($LD_{50}$ of 0.15 μM and 0.7 μM for DTHe and HY respectively). Cell viability after treatment, thus, declined in a dose-dependent manner of both light and photoactivators (Figure 2A and B). No loss of cell viability occurred when the treatments with DTHe or HY were conducted in the absence of light, or when the cells were exposed to light in the absence of the compounds. There was no evidence for non-photodynamic, intrinsic toxicities; cell death resulted from the photodynamic effects of the compounds.

The singlet oxygen quantum yield of HY is 0.42 in ethanol and 0.28 in liposomes, compared with DTHe yields of 0.25 in ethanol and 0.20 in liposomes. Because the singlet oxygen yield of HY is larger than that of DTHe, explanations for the more potent phototoxic activity of the latter were sought in the absorption spectra of the two compounds. These are shown in Figure 3A. The spectra indicate that DTHe has a much broader absorption profile in the visible range that includes the entire range between 450 and 580 nm. Absorption maxima of 3×10$^4$ (ε in dm$^3$ mol$^{-1}$ cm$^{-1}$) are seen at 564 nm, 3.7×10$^4$ at 520 nm and 5.1×10$^4$ at 488 nm. HY has two main absorption peaks at 545 and 590 nm. DTHe, thus, absorbs a larger portion of the total output of a polychromatic light source. The emission spectrum distribution of the source is shown in Figure 3B. Quantitative analyses of the uptake of each compound by HL-60 cells after 1 h of incubation at 37°C revealed no evidence for a larger uptake of DTHe by these cells compared with HY (Figure 4). The amounts of cell-associated HY were approximately 2.5-fold larger than cell-associated DTHe when both compounds were administered at equimolar concentrations (Figure 4).

### The mechanism of photodynamically induced cell death in HL-60 cells

The modes of photodynamically induced HL-60 cell death by HY and DTHe were evaluated by comparative microscopy of cytospin cell preparations. Cells were excited with 0.2–20 μM HY or DTHe (0.5 $\log_{10}$ increments) and 7.2 J cm$^{-2}$ of light. Morphologically normal cells, apoptotic bodies and cells which exhibit features of necrosis were scored as they are shown in Figure 5. The results are shown in Figure 6. At the lower photodynamic dose range of 0.2–2.0 μM DTHe, the prevalent mode of cell death was apoptosis, which, at 0.65 μM DTHe, was the only form of cell death recognizable (Figure 6A). The nuclei were fragmented and the chromatin became condensed with loss of its granular texture. As the levels of photosensitization were increased to ≥6.5 μM DTHe, cell death occurred via an apparent necrosis as shown in Figure 5C. The nuclei were enlarged, basophilic staining was lost and characteristic perinucleolar ring-like condensations of chromatin formed (Figure 5C) that were resistant to further increases in photodynamic damage (data not shown). With HY, formation of apoptotic bodies occurred only at ≤ 0.65 μM with only about 50% of the cells. At higher doses, dying cells exhibited mainly 'necrosis' (Figure 6B). The transition from apoptosis to morphological necrosis occurred abruptly in HY-sensitized cells, at concentrations that were approximately threefold lower than with DTHe.

Flow cytometric analyses were performed concomitantly to assess changes in cellular DNA content. After photosensitization with 0.20–2.00 μM DTHe and 7.2 J cm$^{-2}$ light, the DNA content of the

 © Cancer Research Campaign 1999

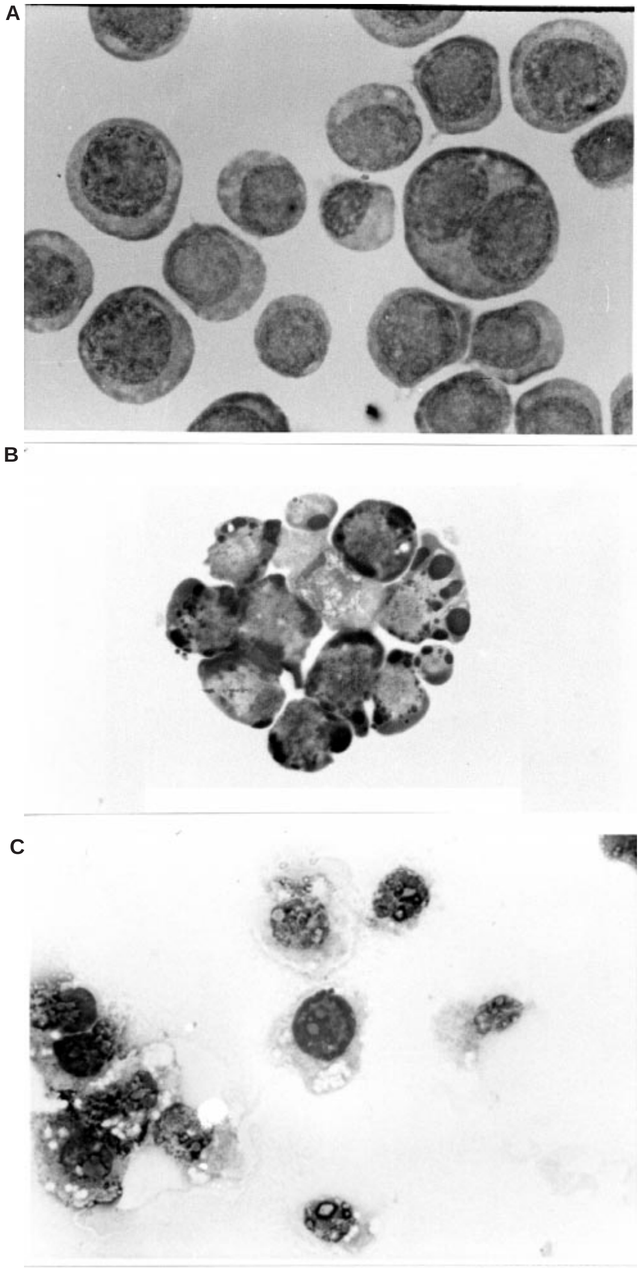

**Figure 5** Effects of different photosensitization doses with DTHe on HL-60 cells (magnification × 700). After photosensitization, all cultures were incubated for 5 h at 37°C, 5% carbon dioxide before staining with May–Grunwald–Giemsa. (**A**) Untreated HL-60 cells. (**B**) Widespread apoptosis obtained with 0.65 µM of DTHe. (**C**) Cell death via necrosis (6.5 µM of DTHe and 7.2 J cm$^{-2}$ of light). Nuclei have become swollen, the chromatin dispersed unevenly, basophilic staining properties were lost and perinucleolar rings of condensed chromatin became evident cells declined (Figure 6C). This was most likely due to fragmentation by endodeoxyribonuclease that occurred when the cells had gone through apoptosis, as evidenced by the DNA ladder formation that occurred after photosensitizing the cells at these doses (Figure 7, lanes 2 and 3). At DTHe doses >2 µM, in which nuclear fragmentation was prevented and the cells exhibited morphological necrosis, the cellular DNA content continued to decline. At high DTHe doses of 6.5 µM only, the extensive photosensitization prevented the decline in cellular DNA content (Figure 6C), possibly due to damage to the endodeoxyribonuclease. DNA fragmentation was completely abrogated at DTHe doses of 20 µM (Figure 7, lane 4). The nuclear fragmentation (formation of morphological apoptotic bodies) was apparently more photosensitive than the DNA ladder-forming endodeoxyribonuclease. Cell death at ≥20 µM of DTHe exhibited only features of necrosis.

### Photodynamic effects on K-562 cells

The photodynamic effects of DTHe and HY on cell death were also evaluated in K-562 cells (Figure 8). The sensitivity thresholds of K-562 cells to DTHe or HY-mediated phototoxicities were close to those of HL-60 cells. Yet, K-562 cells apparently developed fewer morphological apoptotic bodies with nuclear fragmentation in response to photodynamic stimuli with DTHe. A maximum of 50% of the cells became apoptotic at a peak of 0.2 µM and 7.2 J cm$^{-2}$ of light (Figure 8A); the range at which apoptosis has taken place was 0.2–0.65 µM of DTHe. DNA fragmentation to oligonucleosomes was extensive up to 6.5 µM of DTHe (data not shown). With HY as photoactivator, almost no apoptotic bodies were detected at any of the doses applied (Figure 8B) and necrosis was induced at ≥ 0.65 µM. DNA fragmentation to oligonucleosomes in K-562 cells also occurred at doses ≥0.65 µM of HY (data not shown).

### Photodynamic effects on Hut-78 cells

Hut-78 cells were totally resilient to photodynamically induced apoptosis by DTHe or HY. There was no evidence for any apoptosis-related events after photoexcitation with either of the two compounds (data not shown). As the photosensitization exceeded the tolerance threshold, Hut-78 cells died via necrosis. Cellular DNA, monitored on 1.2% agarose gels, remained undigested throughout all photosensitization ranges that were applied with DTHe or HY (data not shown).

### Expression of Bcl-2, Bcl-$X_L$ and Bax proteins in HL-60, K-562 and Hut-78 cells

The firm resistance of Hut-78 cells to photodynamic induction of apoptosis prompted analyses of expression of Bcl-2, Bcl-X and Bax, members of the Bcl-2 gene family which regulate apoptosis, in all three cell lines by Western blots. Fas and tumour necrosis factor (TNF) receptors were analysed by functional assays. Alternative splicing of Bcl-X yields a long, anti-apoptotic transcript Bcl-$X_L$, which acts as an ion channel, or a pro-apoptotic short transcript Bcl-$X_S$ (Boise et al, 1993). The results (Table 1) show no expression of p53 in all three cell lines. Bcl-2 was detected only in HL-60 cells and was absent in K-562 and in Hut-78 cells. Hut-78 cells overexpress Bcl-$X_L$ and a 29-kDa truncated isoform of Bcl-$X_L^{tr}$, which was strongly reactive with an anti-Bcl-X antibody in Western blots (Figure 9A) yet was larger than Bcl-$X_S$. Bcl-$X_L^{tr}$ was only marginally expressed in HL-60 or in K-562 cells (Figure 9A). Overexpression of the combination of Bcl-$X_L$ and Bcl-$X_L^{tr}$ were not evident in any of the cell lines that became apoptotic after photodynamic stress.

The truncated isoform Bcl-$X_L^{tr}$ was found to differ from Bcl-$X_L$ in retainability within photodynamically damaged Hut-78 cells. The concentrations of Bcl-$X_L^{tr}$ and Bax diminished rapidly after photosensitization with DTHe and became barely detectable 3 h after excitation with the highest dose of 6.5 µM (Figure 9B, 3 h). At the 5-h timepoint, these declines were detectable at 2 µM. Bcl-$X_L$

*© Cancer Research Campaign 1999* 

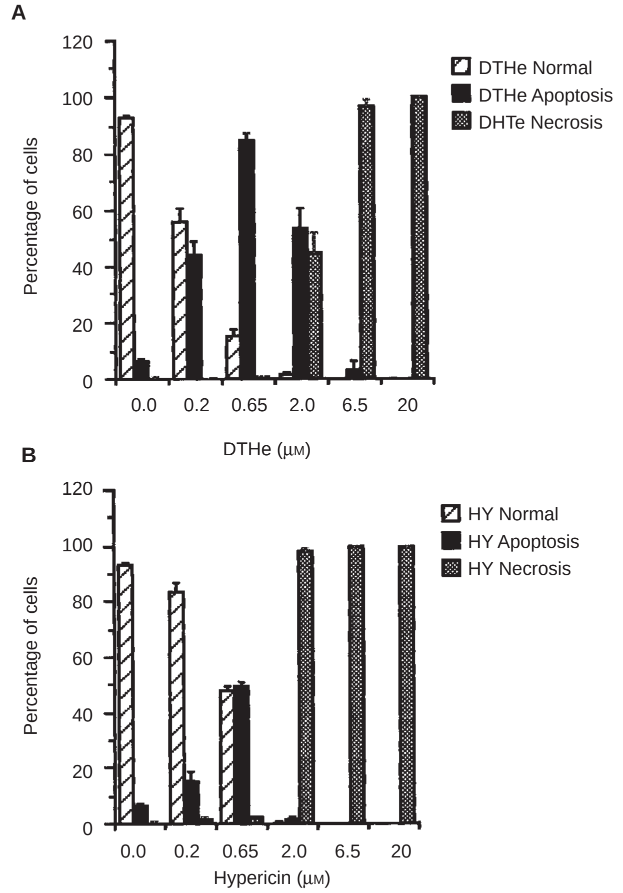

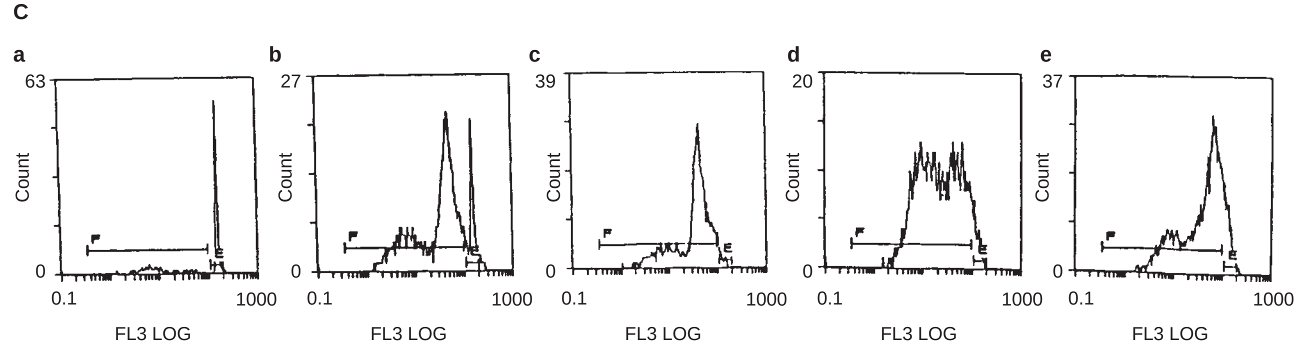

**Figure 6** Microscopic and flow cytometric analyses of the mechanisms of cell death induced by DTHe and by HY in HL-60 cells. Cells photosensitized with DTHe (**A**) or HY (**B**) and 7.2 J cm$^{-2}$ of light were cultured for 5 h. Cytospin preparations were made from 50% of the cells and 4×100 cells were counted in each preparation. The results are given ± standard deviation. The remaining 50% were rinsed with PBS, fixed with 70% ethanol, stained with propidium iodide and analysed for DNA content by flow cytometry (**C**). Graph **a** – untreated HL-60 cells; graphs **b**–**e** show cells photosensitized with 0.2, 0.65, 2 and 6.5 μM DTHe respectively. Region E corresponds to the location of the DNA peak of cells in G0/G1 phase levels remained close to baseline. With HY as the photosensitizer, a similar pattern has been observed (data not shown).

### Effect of photosensitization with DTHe and HY on lamin B polymerization and oligomerization

The collapse of the skeleton of the cell nucleus, which occurs during apoptosis and during mitosis, is elicited by dissociation of laminar filaments (Peter et al 1990). Lamins A, B and C are major constituents of these filaments. Phosphorylation of lamins on serine and threonine residues initiates their dissociation and triggers the disintegration of the filament structures.

We examined the photodynamic effects of DTHe and HY on constituents of the nuclear lamina. Patterns of lamin B dissociation and release from the nucleus into the cytosol after photoexcitation with DTHe or with HY were analysed in Western blots. Figure 10A shows an increase in the cytosolic levels of the 67-kDa lamin B monomer and of an 86-kDa protein that cross-reacts with the antibody after excitation with DTHe (lanes 2–4). The amounts of the 86-kDa polypeptide in the cytosol increased with the dose of the photosensitizer, beginning at 0.65 μM DTHe up to the transition dose from apoptosis to necrosis (6.5 μM DTHe, lane 5). The release of the 67-kDa lamin B from the nuclear envelope into the cytosol also occurred with HY (lanes 6–9), however the 86-kDa protein was not detected in the cytosol of HY-treated cells and its release appeared to be unique to the action of DTHe.

In the nuclear envelope compartment, the photodynamic effects appeared to have prevented the dissociation of lamin polymers into monomeric form. Large molecular weight, covalently bound, photoproducts of 210 kDa were formed, which strongly reacted with anti-lamin antibodies. Their molecular size suggests that they may be lamin B oligomers, possibly trimers of that protein. Their

© Cancer Research Campaign 1999

| Lane | 1 | 2 | 3 | 4 |
|---|---|---|---|---|
| DTHe (μM ml$^{-1}$) | 0 | 0.65 | 6.50 | 20.00 |

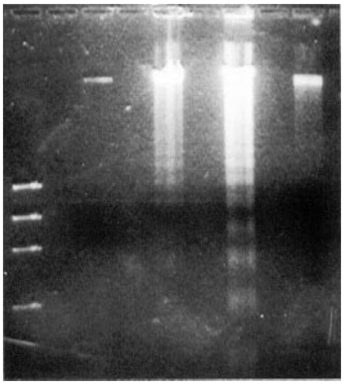

| | | | | |
|---|---|---|---|---|
| % Normal cells | 94 | 14 | 2 | 0 |
| % Apoptotic cells | 5 | 84 | 6 | 0 |
| % Necrotic | 1 | 2 | 92 | 100 |

**Figure 7** Correlation between apoptotic DNA digestion and nuclear fragmentation. Agarose gel electrophoresis of HL-60 cell DNA after photosensitization with the indicated concentrations of DTHe and 7.2 J cm$^{-2}$ light, followed by cultivation for 5 h. Samples were also taken for morphological microscopy. Four hundred cells were counted and the correlating percentages of cells that underwent apoptosis and necrosis are given below

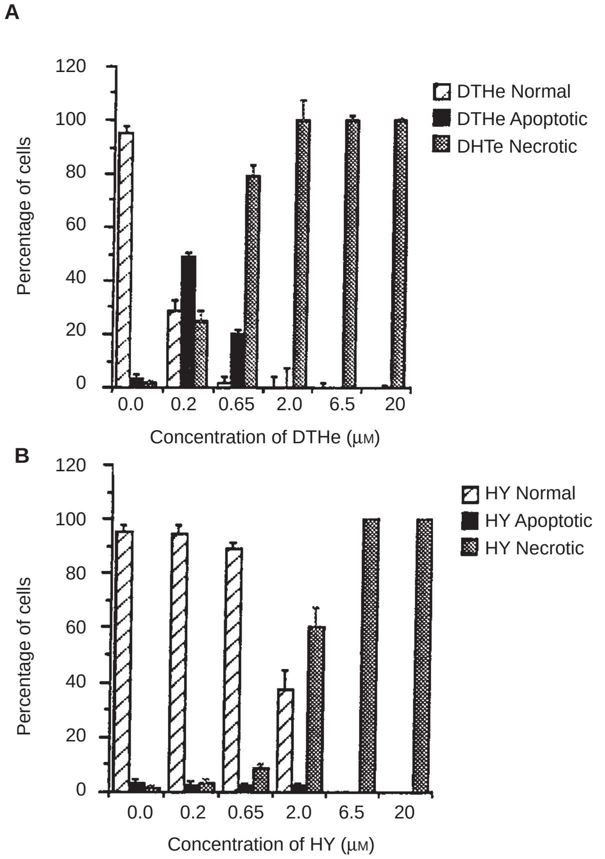

**Figure 8** Microscopic analysis of the mechanisms of cell death induced by DTHe or HY on K-562 cells. Cells were exposed to DTHe (**A**) and to HY (**B**) and 7.2 J cm$^{-2}$ of light followed by 5 h in culture. Cytospin preparations were then prepared, stained with May–Grunwald–Giemsa and counted for normal viable cells, apoptotic figures and cells that exhibit morphological necrosis (± standard deviation)

occurrence coincided with the disappearance of monomeric 67-kDa lamin B. The amounts of the 210-kDa lamin-related photoproducts in the nucleus increased in relation with the photosensitizing doses and peaked at 0.65 μM DTHe (Figure 10B, lane 3). With HY, lamin B-related photoproducts increased at 0.2–0.65 μM (lanes 6 and 7) and were eliminated from the gels at higher photodynamic doses (lanes 8 and 9). Their elimination appeared to have resulted from more extensive polymerization, which prevented entry into the gels and sequestration at the application origin. A portion of the cells that were utilized in the preparation of nuclear extracts were incubated for 5 h, fixed, stained and examined for formation of apoptotic bodies. Nuclear breakdown was apparent in all DTHe samples up to 2 μM (data not shown). Thus, the accumulation of 210-kDa lamin B-reactive photoproducts within nuclei did not interfere with the disintegration of nuclei into small fragments as part of the apoptotic process. Interference with nuclear collapse coincided with the elimination of lamin B from the gels.

### DISCUSSION

The photosensitizing properties of two dianthraquinones were examined: the novel DTHe with a perylene quinone chromophore and absorption spectrum which resembles that of the photodynamically potent hypocrellins (Diwu, 1990, 1995; Miller, 1997) and HY. The mechanisms by which they inflict damage to leukaemic cells were analysed in vitro.

DTHe shares similarities with HY in both chemical structure and lipophilicity. Although HY is a more potent photosensitizer, cell death induction by DTHe, in HL-60 and K-562 cells, occurred at $LD_{50}$ doses three- to fivefold lower than HY under identical lighting conditions. This was, apparently, not an outcome of more extensive cell binding of DTHe compared with HY (Figure 4). One possible explanation for the relatively effective phototoxicity of DTHe may be related to its broader absorption profile in the visible spectrum (Figure 3). The considerable range of light absorption permits DTHe excitation by larger portions of the total emitted output from polychromatic light sources. Thus, the absorption range and utilization of polychromatic sources may also be factors worthy of consideration in devising high-efficiency phototherapeutic systems. Although the long visible range is excluded from DTHe's absorption profile, potentially compromising its efficacy in deep tissues, the broad absorption range might, nevertheless, render DTHe less susceptible to competition for light by tissue porphyrins.

The studies revealed two apparent modes of cell death induced by the photodynamic effects of DTHe that are dose related. At photosensitization levels that immediately exceed the cells tolerance threshold, DTHe induced apoptosis, or programmed cell death, in which cells trigger their own suicide, with all the features of apoptosis: formation of morphological apoptotic bodies, fragmentation of the nuclei, chromatin condensation and DNA digestion to oligonucleosomes (ladder formation). Increases in the dose of the photosensitizer (>2 μM) resulted in inhibition of nuclear fragmentation and the cells acquired a morphology of apparent necrosis. The endodeoxyribonuclease activity, which degrades DNA to oligonucleosomes in apoptosis, proceeded at these higher photosensitizing doses, indicating that it was more photoresistant than the nuclear fragmentation process. All features of apoptosis were subsequently lost ≥20 μM

© Cancer Research Campaign 1999

**Table 1** Expression of proteins that affect pathways that lead to apoptosis

| | HL-60 cells | K-562 cells | HUT-78 cells |
|---|---|---|---|
| Fas/Apo-1R[a] | + | – | + |
| TNF-R[b] | + | + | – |
| Bcl-2 | + | – | – |
| Bcl-$X_L$ | + | ++ | +++ |
| Bax | + | + | + |
| p53 | – | – | – |

[a]Fas/Apo-1R was monitored by double antibody immunostaining and FACS analysis, as well as by induction of apoptosis by an anti-Fas monoclonal antibody scoring the percentage of apoptotic bodies. [b]TNF-R was monitored by induction of apoptosis by 1 nM TNF-α in combination with cycloheximide (1 μg ml$^{-1}$). Expression of Bcl-2, Bcl-x, Bax and p53 were analysed by Western blots.

DTHe, with cessation of DNA digestion in a manner similar to that described with porphyrins (Noodt et al, 1996). The transition from viable cells to apoptotic bodies was associated with a 0.5 $\log_{10}$ reduction in DNA fluorescence analysed by FACS, which coincided with partial DNA digestion to oligonucleosomes (Figure 7, lane 2). Further increases in the dose of DTHe, which resulted in morphological cell necrosis, were associated with another 0.5 $\log_{10}$ reduction in DNA fluorescence (Figure 6C, d), apparently an outcome of more extensive DNA digestion to oligonucleosomes (Figure 7, lane 3). At photosensitization levels ≥ 6.5 μM and 7.2 J cm$^{-2}$ of light, the cellular DNA content was higher than in cells that received lower doses of photosensitization by 0.5 $\log_{10}$, suggesting that damage to the endonuclease diminished DNA digestion. The electrophoretic patterns of DNA digestion in agarose gels of DTHe-treated HL-60 cells support this hypothesis (Figure 7, lane 4). In Hut-78 cells, death occurred exclusively via necrosis with neither DNA nor nuclear fragmentation evident at any photosensitizing level (data not shown).

The photodynamic effects of DTHe or HY were considerably different. DTHe induced primarily apoptosis at a rather broad photosensitization range. In HL-60 cells, morphological apoptosis shifted to apparent necrosis only at doses tenfold higher than the minimal toxic dose. HY induced more necrosis; fewer HL-60 apoptotic bodies were formed at narrower photosensitizing ranges (Figure 5), and only sporadic apoptotic figures were detected in K-562 cells. An incomplete apoptosis by HY, characterized by DNA degradation to oligonucleosomes (ladder) in the absence of nuclear fragmentation, also occurs in murine mammary carcinoma (Thomas and Pardini, 1992b) and in neuroblastoma cells (Zhang et al, 1995).

The apparent necrosis was associated with condensation of chromatin to form rings around the nucleoli in all cell lines. A chain of globular chromatin, visible shortly after photosensitization, condensed into a homogeneous ring after treatments with high levels of photodynamic stress. These rings were photoresistant and developed at the highest photosensitization levels applied (14.4 J cm$^{-2}$).

The prevention of nuclear disintegration after photodynamic induction of apoptotic cell death has alternative possible explanations. One is related to the documented inhibition of PKC activity by HY, particularly when the enzyme translocates to the surface membrane of cells (Takahashi, 1989; Agostinis, 1995). Protein kinase C $\beta_{II}$ is an enzyme that translocates to the nuclear membrane where, together with $p34^{cdc2}$ kinase (Peter et al, 1990), it phosphorylates lamins on serine/threonine residues (Murray et al, 1994). The phosphorylation disassembles lamin filaments and

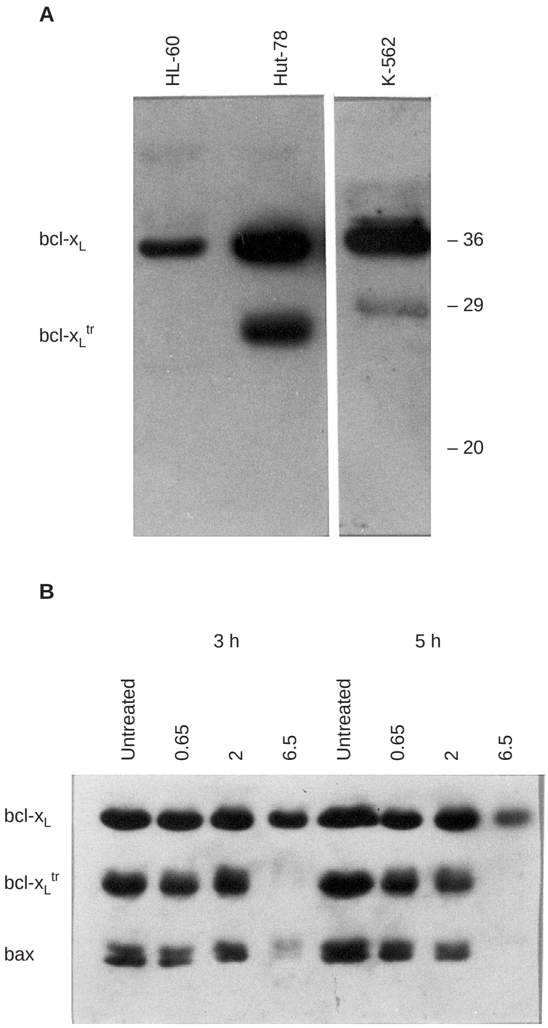

**Figure 9** Bcl-x expression in Hut-78 cells by Western blots.(**A**) Cytosolic fractions of Hut-78 cells were loaded onto a 12.5% SDS-PAGE gel, 30 μg protein per well, and analysed for reaction with anti-Bcl-x antibody in comparison with HL-60 and K-562 cells. The gel was transblotted to a nitrocellulose filter, reacted with anti-Bcl-x antibody and developed for chemiluminescence. (**B**) Expression of Bcl-x isoforms and Bax after photodynamic excitation with DTHe at the concentrations indicated and light irradiance of 7.2 J cm$^{-2}$

manifests nuclear membrane breakdown during apoptosis (Kick et al, 1996) and also mitosis. Lamins also appear to be degraded by caspases during apoptosis (Takahashi et al, 1996) and photosensitization induces the cleavage and activation of caspase 3 (Granville et al, 1997). PKC $\beta_{II}$ might be a potential target for light-dependent inhibition by HY, with consequent interference with lamin phosphorylation, stabilization of the nucleus and prevention of nuclear fragmentation. This model could explain the transition from apoptosis to apparent necrosis with the increase in photodynamic damage to the cells. The broader photosensitization range at which apoptosis occurs with DTHe might suggest a lesser PKC inhibitory activity on the part of DTHe.

An alternative mechanism to explain the inhibition of nuclear disintegration may be photodynamic cross-linking of lamins. Evidence for possible photodynamically generated lamin cross-linking in HL-60 cells, treated with DTHe or HY, is shown in Figure 10B. Large, covalently linked polypeptides that react with anti-lamin B antibodies formed on Western blots. Photo-oxidation by

 © Cancer Research Campaign 1999

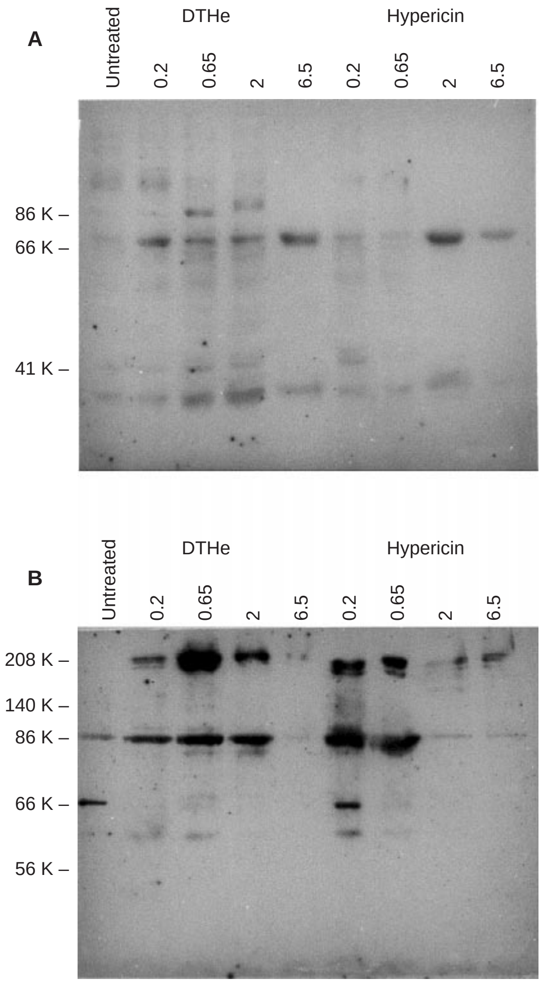

**Figure 10** Lamin B configurations in the cytosol (**A**) and in the nuclear envelope (**B**) of HL-60 cells after photosensitization with DTHe or HY. HL-60 cells were excited with 0.1–3.3 μg ml$^{-1}$ DTHe or HY (corresponding to approximately 0.2–6.5 μM) and 7.2 J cm$^{-2}$ of light. The cells were incubated at 37°C for 3 h, lysed in 1% Triton X-100 in Tris buffer; the cytosol was separated, run on SDS-PAGE and transblotted with an anti-lamin B1 antibody (**A**). The nuclei were pelleted by centrifugation at 6500 ***g*** and washed once with PBS containing 5 mM magnesium chloride. The nuclei were then dissociated directly into Laemmli buffer without any specific lamin extraction procedure, separated on a 10% SDS polyacrylamide gel, transblotted onto a nitrocellulose filter, reacted with anti-human lamin B1 antibody and developed by enhanced chemiluminescence (ECL) (**B**)

HY has been shown to cross-link covalently a variety of other proteins (Senthil et al, 1992), and excited oxygen species can affect lamins in a similar manner. The 210-kDa photoproduct occurred in DTHe-treated cells that have gone through a complete process of apoptosis, including fragmentation of their nuclei. Thus, its formation at low photosensitization doses did not interfere with nuclear breakdown and completion of apoptosis. Higher photosensitizing doses appeared to have yielded more extensive lamin cross-linking with formation of non-dissociable lattices, which were incapable of entering SDS gels and may have prevented nuclear collapse. Indeed, at these highest doses, the amounts of the 210-kDa polypeptides in the gels decreased. It may be noteworthy that a similar mechanism elicits the virucidal activity of HY to retroviruses. Covalent cross-linking of retroviral capsid proteins were found to create lattices that prevented capsid unfolding during de novo cell infection (Degar et al, 1992) and release of the reverse transcriptase enzyme and the viral RNA genome from retrovirions (Lavie et al, 1989).

Necrosis, in which the cell-surface membrane is disrupted and the intracellular contents are discharged into the extracellular environment, is associated with inflammation. Both HY and DTHe elicit tumour cell necrosis at high photodynamic doses. The associated inflammatory responses are considered to be beneficial to systemic antitumoral effects; for example, BCG has proven useful in treating some forms of cancer, such as melanoma and transitional carcinoma of the bladder. Photosensitization enhances production of cytokines such as TNF and interleukin 6 (IL-6) (Evans et al, 1990; Kick et al, 1995), which play important antitumoral and antimetastatic roles. With HY, photosensitization-related side-effects can be quite severe and are termed hypericism (Pace, 1942). DTHe, being an effective phototoxic agent with milder effects of necrosis, is anticipated to be better tolerated in PDT of tumours in vivo.

These studies of the photodynamic effects of dianthraquinones on human leukaemic cells appear to identify a unique apoptotic pathway. Photosensitized HL-60 and K-562 cells die by apoptosis. Hut-78 cells, however, were firmly resistant to apoptosis via that pathway, but not to apoptosis induced with an anti-Fas antibody [illegible] pathway appears to be p53 independent, and can be inhibited by mechanisms independent of overexpression of the survival promoting Bcl-2. HL-60 cells that express Bcl-2 became apoptotic after photosensitization with DTHe, whereas Hut-78 cells, which express no Bcl-2, were highly resistant to it. Hut-78 cells expressed high levels of Bcl-$X_L$ and a 29-kDa truncated isoform that was strongly reactive with anti-Bcl-X antibody raised against a peptide derived from N-terminal amino acid residues 2–19. High expressions of the two were not evident in cells that became apoptotic after photodynamic treatment. The truncated Bcl-$X_L^{tr}$ was only marginally expressed in HL-60 or in K-562 cells (Figure 9A). Bcl-$X_L$ or its truncated form may have prevented the photodynamic induction of apoptosis in Hut-78 cells. Bcl-$X_L$ and Bcl-2 can regulate ion flux channels and Bcl-2 has been suggested to inhibit apoptosis by interfering with cytochrome *c* and $Ca^{2+}$ release into the cytoplasm (Kluck et al, 1997; Yang et al, 1997). Bcl-$X_L^{tr}$ also differed from Bcl-$X_L$ in retainability within photodynamically damaged cells. Bcl-$X_L^{tr}$ and Bax levels diminished rapidly after cell photosensitization at high doses that caused necrosis. Bcl-$X_L$ was better retained, possibly because of its membrane association. Bcl-$X_L^{tr}$ and Bax might have either leaked out of the cells because of absence of membrane anchoring, or were more sensitive to photodynamic damage.

Altogether, these studies suggest that DTHe and the related HY offer photodynamic properties that, in association with polychromatic light, may render them suitable for further development as second-generation mediators of photodynamic therapy.

### ACKNOWLEDGEMENTS

The authors are grateful to Dr Joseph Lotem, Department of Molecular Genetics, The Weizmann Institute of Science, Israel, for a critical review of this manuscript. The study was supported by NIH grant no. HL 53379-01 and by grant no. 3181 of the Israeli Health Ministry.

### REFERENCES

Agostinis P, Vandenbogaerde A, Donella-Deana A, Pinna LA, Lee K, Goris J, Merlevede W, Vandenheede JR and de Witte P (1995) Photosensitized inhibition of growth factor-regulated protein kinases by hypericin. *Biochem Pharmacol* **49**: 1615–1622

Agostinis P, Donella-Deana A, Cuveele J, Vandenbogaerde A, Sarno S, Merlevede W and de Witte P et al (1996) A comparative analysis of the photosensitized inhibition of growth-factor regulated protein kinases by hypericin derivatives. *Biochem Biophys Res Commun* **220**: 613–617

Anker L, Gopalakrishna R, Jones KD, Antel JP, Apuzzo MLJ and Couldwell WT (1995) Hypericin in adjuvant brain tumor therapy. *Drugs Future* **20**: 511–517

Boise LH, Gonzalez-Garcia M, Postema CE, Ding L, Lindsten T, Turka LA, Mao X, Nunez G and Thompson CB (1993) Bcl-X, a bcl-2-related gene that functions as a dominant regulator of apoptotic cell death. *Cell* **74**: 597–608

Carpenter S and Kraus GA (1991) Photosensitization is required for inactivation of equine infectious anemia virus by hypericin. *Photochem Photobiol* **53**: 169–174

Chattopadhyay SV, Kumar CV and Das PK (1984) Laser flash photolytic determination of triplet yields via singlet oxygen generation. *J Photochem* **24**: 1–9

Chung PS, Saxton RE, Paiva MB, Rhee CK, Soudant J, Mathey A, Foote C and Castro DJ (1994) Hypericin uptake in rabbits and nude mice transplanted with human squamous cell carcinoma: study of a new sensitizer for laser phototherapy. *Laryngoscope* **104**: 1471–1476

Degar S, Prince AM, Pascual D, Lavie G, Levin B, Mazur Y, Lavie D, Ehrlich LS, Carter C and Meruelo D (1992) Inactivation of the human immunodeficiency virus by hypericin: Evidence for photochemical alterations of p24 and a block in uncoating. *AIDS Res and Human Retroviruses* **8**: 1929–1936

Degar S, Lavie G, Meruelo D (1993) Photodynamic inactivation of Radiation Leukemia virus produced from hypericin treated cells. *Virology* **197**: 796–800

Diwu Z (1995) Novel therapeutic and diagnostic applications of hypocrellins and hypericin. *Photochem Photobiol* **61**: 529–539

Diwu Z and Lown JW (1990) Hypocrellins and their use in photosensitization. *Photochem Photobiol* **52**: 606–616

Diwu Z and Lown JW (1993) Photosensitization with anticancer agents 17. EPR studies of photodynamic action of hypericin: formation of semiquinone radical and activated oxygen species on illumination. *Free Radical Biol Med* **14**: 209–215

Dougherty TJ (1983) Hematoporphyrin as a photosensitizer of tumors. *Photochem Photobiol* **38**: 377–379

Evans S, Matthews W, Perry R, Flaker D, Norton J and Pass HI (1990) Effect of photodynamic therapy on tumor necrosis factor production by murine macrophages. *J Natl Cancer Inst* **82**: 34–39

Gomer CI (1991) Preclinical examination of first and second generation photosensitizers used in photodynamic therapy. *Photochem Photobiol* **61**: 529–539

Granville DJ, Levy JG and Hunt DWC (1997) Photodynamic therapy induces caspase-3 activation in HL-60 cells. *Cell Death Differ* **4**: 623–626

Grossweiner LI (1994) Photodynamic therapy. In *The Science of Phototherapy*, Chapter 8. CRC Press: Boca Raton, FL

Hadjur C, Jeunet A and Jardon P (1994) Photosensitization by hypericin: ESR evidence for singlet oxygen and superoxide anion radicals formation in an in vitro model. *J Photochem Photobiol B Biol* **26**: 67–74

Hadjur C, Richard MJ, Parat MO, Favier A and Jardon P (1995) Photodynamically induced cytotoxicity of hypericin dye on human fibroblast cell line MRC5. *J Photochem Photobiol* **27**: 139–146

Honigsmann H, Tanew A and Wolff K (1987) Treatment of mycosis fungoides with PUVA. *Photo Dermatol* **4**: 55–58

Hudson JB, Harris L and Towers GHN (1993) The importance of light in the anti-HIV effect of hypericin. *Antiviral Res* **20**: 173–178

Jones LR and Grossweiner LI (1996) Effect of Photofrin R on in vivo skin reflectivity. *J Photochem Photobiol* **B33**: 153–156

Kessel D (1984) Hematoporphyrin and HPD, photophysics, photochemistry and phototherapy. *Photochem Photobiol* **39**: 851–859

Kick G, Messer G, Goetz A, Plewig G and Kind P (1995) Photodynamic therapy induces expression of interleukin 6 by activation of AP-1 but not NF-κB DNA binding. *Cancer Res* **55**: 2373–2379

Kick G, Messer G, Plewig G, Kind P and Goetz A (1996) Strong and prolonged induction of c-*jun* and c-*fos* proto-oncogenes by photodynamic therapy. *Br J Cancer* **74**: 30–36

Kluck RM, Bossy-Wetzel E, Green DR and Newmeyer DD (1997) The release of cytochrome c from mitochondria: a primary site for Bcl-2 regulation of apoptosis. *Science* **275**: 1132–1136

Lavie G, Valentine F, Levin B, Mazur Y, Gallo G, Lavie D, Weiner D and Meruelo D (1989) Studies of the mechanisms of action of the antiretroviral agents hypericin and pseudohypericin. *Proc Natl Acad Sci USA* **86**: 5963–5967

Lavie D, Freeman D, Bock H, Fleischer J, van Kranenburg K, Ittah Y, Mazur Y, Lavie G, Liebes L and Meruelo D (1990) Hypericin, a potential anti-AIDS drug. In *Trends in Medicinal Chemistry '90. Proceedings of the XIth International Symposium on Medicinal Chemistry, 2–7* September, *Jerusalem, Israel*, pp. 321–327

Li X, Traganos F and Darzynkiewicz Z (1994) Simultaneous analysis of DNA replication and apoptosis during treatment of HL-60 cells with camptothecin and hyperthermia and mitogen stimulation of human lymphocytes. *Cancer Res* **54**: 4289–4293

Lotem J and Sachs L (1995) Regulation of bcl-2, bcl-x and bax in the control of apoptosis by hematopoietic cytokines and dexamethasone. *Cell Growth Difference* **6**: 647–653

Lotem J, Cragoe EJ and Sachs L (1991) Rescue from programmed cell death in leukemic and normal myeloid cells. *Blood* **78**: 953–960

McCaughan Jr JS (1984) Photoradiation of malignant tumors presensitized with hematoporphyrin derivative. *Prog Clin Biol Res* **170**: 805–827

Meruelo D, Lavie G and Lavie D (1988) Therapeutic agents with dramatic antiretroviral activity and little toxicity at effective doses: aromatic polycyclic diones hypericin and pseudohypericin. *Proc Natl Acad Sci USA* **85**: 5230–5234

Miller GG, Brown K, Ballangrud AM (1997) Preclinical assessment of hypocrellins and hypocrellin B derivatives as sensitizers for photodynamic therapy of cancer: progress update. *Photochem Photobiol* **65**: 714–722

Mossman T (1983) Rapid colorimetric assay for cellular growth and survival: application to proliferation and cytoxicity assays. *J Immunogenet* **21**: 235

Murray NR, Burns DJ and Fields AP (1994) Presence of a $\beta_{||}$ protein kinase C-selective nuclear membrane activation factor in human leukemia cells. *J Biol Chem* **269**: 21385–21390

Noodt BB, Berg K, Stokke T, Peng Q and Nesland JM (1996) Apoptosis and necrosis induced with light and 5-aminolaevulinic acid-derived protoporphyrin IX. *Br J Cancer* **74**: 22–29

Orenstein A, Kostenich G, Roitman L, Tsur H, Katanik D, Kopolovic J, Ehrenberg B and Malik Z (1996) Photodynamic therapy of malignant lesions of the skin mediated by topical application of 5-aminolevulinic acid in combination with DMSO and EDTA. *Lasers Life Sci* **7**: 1–9

Pace N (1942) The etiology of hypericism, a photosensitivity produced by St. Johns wort. *Am J Physiol* **136**: 650–656

Peter M, Nakagawa J, Doree M, Labbe JC and Nigg EA (1990) In vitro disassembly of the nuclear lamina and M phase-specific phosphorylation of lamins by cdc2 kinase. *Cell* **61**: 591–602

Senthil V, Longworth JW Ghiron CA and Grossweiner LI (1992) Photosensitization of aqueous model systems by hypericin. *Biochim Biophys Acta* **1115**: 192–200

Takahashi I, Nakanishi S, Kobayashi E, Nakano H, Suzuki K and Tanaoki T (1989) Hypericin and pseudohypericin specifically inhibit protein kinase C: possible relation to their antiretroviral activity. *Biochem. Biophys. Res Commun* **165**: 1207–1212

Takahashi A, Alnemri ES, Lazebnik YA, Fernandes-Alnemri T, Litwack G, Moir RD, Goldman RD, Poilier GG, Kaufmann SH and Earnshaw WC (1996) Cleavage of lamin A by Mch2α but not CPP32: Multiple interleukin 1β-converting enzyme-related proteases with distinct substrate specificities. *Proc Nat Acad Sci USA* **93**: 8395–8400

Tang J, Colacino JM, Larsen SH and Spitzer W (1990) Virucidal activity of hypericin against enveloped and non-enveloped DNA and RNA viruses. *Antiviral Res* **13**: 313–326

Thomas C, MacGill RS, Miller GC and Pardini RS (1992*a*) Photoactivation of hypericin generates singlet oxygen in mitochondria and inhibits succinoxidase. *Photochem Photobiol* **55**: 47–53

Thomas C and Pardini RS (1992*b*) Oxygen dependence of hypericin-induced phototoxicity to EMT6 mouse mammary carcinoma cells. *Photochem Photobiol* **55**: 831–837

Weiner L and Mazur Y (1992) EPR studies of hypericin. Photogeneration of free radicals and superoxide. *J Chem Soc Perkin Trans* **2**: 1439–1442

Yang J, Liu X, Bhalla K, Kim CN, Ibrado AM, Cai J, Peng TI, Jones DP and Wang X (1997) Prevention of apoptosis by Bcl-2: Release of cytochrome c from mitochondria blocked. *Science* **275**: 1129–1132

Zhang W, Lawa RE, Hintona DR, Su Y and Cauldwell WT (1995) Growth inhibition and apoptosis in human neuroblastoma SK-N-SH cells induced by hypericin, a potent inhibitor of protein kinase C. *Cancer Lett* **96**: 31–35

### ADDENDUM

Analytical data for DTHe: $^1$H-NMR, $(CD_3)_2$ CO, δ18,51 (s, 1H), 16,08 (s,2H). 8.34, 8.32 (d,2H, J=8 Hz).7,71 (s,2H) 7.41, 7, 37 (d,2H, J = 8). 6.40 (s,2H), 2.09 (s, 6H) ppm. UV-vis. absorb. spectra (Figure 3): λ 564, 520, 488, 380 nm (ε, $2.5 \times 10^4$, $27 \times 10^4$, $37 \times 10^4$, $35 \times 10^4$, $dcm^3\ mol^{-1}\ cm^{-1}$).

 *© Cancer Research Campaign 1999*
